# Conference Reports: SECOND INTERNATIONAL CONFERENCE ON CHEMICAL KINETICS Gaithersburg, MD July 24–27, 1989

**DOI:** 10.6028/jres.095.024

**Published:** 1990

**Authors:** John T. Herron, Wing Tsang

**Affiliations:** Chemical Kinetics Division, Center for Chemical Technology, National Institute of Standards and Technology, Gaithersburg, MD 20899

The conference, chaired by John T Herron of the Chemical Kinetics Division of NIST, was attended by 240 scientists from 14 countries representing academic, government and private sector institutions.

The conference was sponsored by the National Institute of Standards and Technology, the National Science Foundation, the National Aeronautics and Space Administration, the Environmental Protection Agency, the Gas Research Institute, the Petroleum Research Fund administered by the American Chemical Society, the Ford Motor Company, and the Exxon Research and Engineering Company. The range of the sponsors is indicative of the broad range of interests and applications which were the subjects of the conference.

## 1. The Conference

The conference followed the tradition of previous conferences held at the National Institute of Standards and Technology (formerly National Bureau of Standards) on important issues in chemical kinetics. The focus of these conferences has been on the theory and practice of chemical kinetics with emphasis on its application to important practical and societal problems of national and international scope such as photochemical smog, the antarctic ozone hole, and the use of plasmas to fabricate electronic devices. Thus, this year’s conference had as its subtitle, “Application of Fundamental Data.” That theme was developed in a series of seven conference sessions each focusing on a different aspect of chemical kinetics and its application.

The format of the conference was designed to encourage the broadest possible participation of the kinetics community, while at the same time allowing for a detailed discussion of both oral and poster presentations.

## 2. Introductory Session

The first session was devoted to a series of invited lectures on selected topics in basic and applied chemical kinetics. It was chaired by S. H. Bauer of Cornell University. The speakers and their topics were: S. R. Leone, National Institute of Standards and Technology, “Time-Resolved FTIR Emission Studies of Photochemical Kinetics”; P. Gray, Gonville and Caius College, Cambridge University, “Modeling Complex Behavior by Simple Chemical Schemes”; D. G. Truhlar, University of Minnesota, “Recent Advances in the Calculation of Bimolecular Reaction Dynamics”; S. E. Stein, National Institute of Standards and Technology, “Chemical Databases for Kinetics.” This session illustrated the great advances made in theory and experiment in the past decade, the complexity of chemical systems now under study, and the promise of new means to compile and distribute chemical kinetic data.

## 3. Topical Sessions

Six sessions followed, each opening with three invited talks followed by a poster session and an extended discussion period devoted to all aspects of the materials presented at the session. The discussion periods were led by the session chairmen with the invited speakers playing a major contributory role. The session titles, chairmen, and speakers were:

Session 2, Environmental Chemistry—The Stratosphere

Chairman, M. J. Kurylo, National Institute of Standards and Technology; F.S. Rowland, University of California Irvine, “The Chemistry of Halocarbons and Hydrocarbons in the Atmosphere”; J. G. Anderson, Harvard University, “Free Radicals in the Antarctic and Arctic Vortices: Ozone Destruction in the Polar Stratosphere”; M. Tolbert, SRI International, “Laboratory Studies of Heterogeneous Processes in the Stratosphere.” This session provided a remarkable overview of the current status of our knowledge of the consequences of introducing halogen containing sub-stances into the atmosphere. Laboratory studies are now beginning to provide a basis for understanding and ultimately predicting the anomalous ozone levels observed in the antarctic and arctic atmospheres. The recognition that heterogeneous chemistry plays a major role has been supported by recent experimental work.

Session 3, Environmental Chemistry—The Troposphere

Chairman, D. M. Golden, SRI International; R. A. Cox, Harwell Laboratory, “Laboratory Studies of Peroxy Radical Reactions of Importance for Tropospheric Chemistry”; H. Niki, York University, “Fourier Transform Infrared Spectroscopic Studies of Atmospheric Reactions involving Hydrocarbons”; W. L. Chameides, Georgia Institute of Technology, “Urban Photochemical Smog: The Problems, Uncertainties, and Possible Solutions.” As this session illustrated, there remain many fundamental unresolved problems in tropospheric chemistry with very broad societal implications. The chemical kinetics mechanisms are still not well understood, nor is the role of natural hydrocarbon sources. The basis of current control strategies was also challenged.

Session 4, Ions, Clusters and Interfaces

Chairman, P. Davidovits, Boston College; A. W. Castleman, Jr., Pennsylvania State University, “Cluster Reactions”; C. E. Kolb, Aerodyne Research, Inc., “Sticking of Gaseous Molecules on Liquid Droplets—Applications to Atmospheric Acid Deposition and Ozone Depletion.” This session further emphasized the recognition that interfacial chemistry plays a crucial role in many aspects of atmospheric chemistry, ranging from the depletion of ozone in the antarctic to tropospheric processes of acid deposition. The advances in measurement science are very apparent in these areas.

Session 5, Plasma Chemistry

Chairman, G. Hancock, Oxford University; D. L. Flamm, University of California and AT&T Bell Laboratories, “Kinetics of Silicon Etching in Fluorine and Chlorine Containing Plasmas”; J. W. Hudgens, National Institute of Standards and Technology, “The Observation of Silicon Centered Free Radicals Using Multiphoton Ionization Spectroscopy”; J. M. Jasinski, The Thomas J. Watson Research Center, IBM, “Silane Plasma Chemistry One Step at a Time: Silicon Hydride Radical-Molecule Kinetics.” Plasma chemistry is now entering a new era in which modeling of complex processes on the basis of elementary chemical reactions is being actively pursued. This session emphasized the range of plasma chemistry under study, some of the highly specific diagnostic approaches being developed, and basic chemical kinetic measurement and modeling activities being carried out. The potential for advances in this previously unexplored area of chemical kinetics promises to be of great practical importance and almost certainly will provide new insights into fundamental aspects of chemical kinetics.

Session 6, Oxidation and Combustion

Chairman, A. Lifshitz, Hebrew University; J. Wolfrum, University of Heidelberg, “Elementary Chemical Reactions and Their Interaction with Transport Processes: Experiments and Models”; F. L. Dryer, Princeton University, “Chemical Kinetic Modeling for Combustion Processes”; A. M. Dean, Exxon Research and Engineering Company, “The Role of Chemical Activation in Combustion Chemistry.” Chemical kinetic modeling plays a dominant role in the elucidation of combustion chemistry. In this session the connection was strongly drawn between measurement of the kinetics of elementary reactions at the most elementary level, and processes occurring in practical combustion devices.

Session 7, Propellants and High Energy Chemistry

Chairman, Th. Just, DFVLR; C. F. Melius, Sandia National Laboratories, “The Chemistry of Nitramine Propellant Combustion”; M. C. Lin, Emory University, “Elementary Processes of Relevance to Nitroalkane, Nitramine and Hydrocarbon Combustion Reactions,” J. Troe, University of Gottingen, “From Laser Photolysis Experiments to Thermal Dissociation Rates.” A theoretical basis now exists for the general chemical processes relevant to propellent chemistry. This session outlined that basic conceptual approach, and showed how new laboratory programs are providing the chemical kinetic data base required to corroborate, modify, or extend our knowledge of propellent and high energy chemistry.

## 4. Summary

The emphasis on application meant that the main focus of the conference was on the measurement, estimation, and evaluation of thermal rate constants. The nature of the topics chosen for the technical sessions illustrates the direct contribution that fundamental chemical data can make to the solution of important national issues. In the case of stratospheric chemistry this is particularly well understood and chemical kinetic data are used with great effect. This successful area of application serves to illustrate the enormous potential for applying fundamental data to other practical problems. The tenor of the invited and contributed papers, which provide a catalogue of needs and a demonstration of capabilities, clearly indicates that other areas are ripe for such an organized approach to scientific problem solving. Furthermore, the fundamental nature of chemical kinetics makes advances in one subfield, whether in instrumentation, method development, or theory, immediately applicable to other problem areas.

Looking back at the conference held 4 years ago, it is apparent that there has been a great deal of progress. Nevertheless, as capabilities have improved, so have the needs for reliable kinetic data. There is a sense that many opportunities are not being fully exploited. Part of the problem is undoubtedly due to the quantity of data needed to simulate accurately a complex system. Although advances and applications in theory have made notable contributions during the past 4 years, the need for high quality experimental data will continue to dominate modeling efforts. The experience gained from the stratospheric ozone problem clearly indicates how such measurements contribute not only to the problem at hand, but also to all aspects of usable chemical kinetics. Certainly some of the most exciting developments in chemical kinetics in recent years have sprung from such studies, and in the next four years, we expect to see great advances in the understanding and control of other complex systems in which chemical kinetics plays a controlling role.

Copies of the meeting abstract book are available from the conference chairman. In addition, a special issue of the Journal of Physical Chemistry will be devoted to the conference proceedings.

